# Effect of two postharvest technologies on the micronutrient profile of cashew kernels from Mozambique

**DOI:** 10.1002/fsn3.2658

**Published:** 2021-11-23

**Authors:** Americo Uaciquete, Neid Ali Ferreira, Katja Lehnert, Walter Vetter, Nadine Sus, Wolfgang Stuetz

**Affiliations:** ^1^ Instituto de Investigação Agraria de Mozambique IIAM Centro Zonal Nordeste Posto Agronómico de Nampula Nampula Mozambique; ^2^ Institute of Food Chemistry University of Hohenheim Stuttgart Germany; ^3^ Institute of Nutritional Sciences University of Hohenheim Stuttgart Germany

**Keywords:** cashew kernels, industrial kernel grades, micronutrient profiles, nut drying

## Abstract

The economics involved in processing cashew nuts (*Anacardium occidentale*) might alter micronutrient profiles and concentrations. We analyzed and evaluated carotenoids, tocopherols, tocotrienols, minerals, fatty acids, and amino acids in (1) cashew kernels with testa recovered from nuts dried with and without the apple, and (2) testa‐free industrial grade baby butts, splits, and white whole kernels using HPLC, ICP‐OES, and GC‐MS techniques. The results indicated that drying cashews with the respective apple slightly decreased the concentration of some carotenoids and total fatty and amino acids, but increased the concentration of iron, magnesium, and total tocotrienols compared with the conventionally (sun‐) dried kernels. We also found high concentrations of carotenoids in the testa‐containing kernels. Among the industrially processed kernel, baby butt grade was associated with lower content of β‐carotene, total tocopherols, and tocotrienols, but with significantly higher concentrations in minerals, fatty acids, and amino acids than in white wholes and split grades. Conventional sun drying of cashew nuts revealed results similar to drying with apples regarding micronutrient concentrations. The high micronutrient content of industrial grade BB is reflected in widespread human consumption and better market value.

## Graphical abstract

Impact of nut drying technologies on carotenoids, tocopherols, tocotrienols, minerals, fatty acids and amino acids in cashew kernels was investigated. Higher concentrations of the carotenoids lutein and β‐carotene in the testa‐containing kernels were confirmed. Higher concentrations of minerals, fatty acids and some amino acids were found in baby butts than in whole white and split grades, underlining the nutritional value and possible better market value of the baby butt grades.

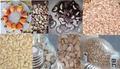

## 1 INTRODUCTION

Cashew (*Anacardium occidentale* L.) is native to tropical America, specifically in Brazil (Bhoomika & Sudha, 2018; Liaotrakoon et al., 2016) and thrives well in tropical climates between 27° North and 28^o^ South of the equator (Griffin & Dean, 2017). Currently, the species is well established in many intertropical countries such as Ivory Coast, Vietnam, India, Tanzania, Nigeria, Mozambique, and others (Mog et al., 2018). The richness in proteins (21%), carbohydrates (25%), monounsaturated fatty acids (48%), minerals, vitamin B, and tocopherols bears testimony to the economic and social importance of cashew kernels on a global scale (Bhoomika & Sudha, 2018; Chung et al., 2013; Olga et al., 2018; Olubode et al., 2020; Rico et al., 2015; Stuetz et al., 2017).

From harvest to consumption, cashew nuts are subjected to a number of technological processes (Akujobi et al., 2018) that include drying, storage, roasting or steaming, deshelling, peeling, and then secondary processing that includes salting, frying, grinding, cooking, and flavoring (Griffin & Dean, 2017; Lima et al., 2015), with the aim of satisfying consumer preferences such as color, size, palatability, flavor, taste, and aroma (Gadani et al., 2017; Lima et al., 2015).

Different processing technologies may result in significantly different nutritional profiles of cashew kernels and products as observed in terms of proteins (17.50% versus 24.49% dry weight) and lipids (39.88% versus 45.40% dry weight) (Lima et al., 2015). Maize and cashew kernels technologically processed into fermented, germinated, and roasted flour were found to improve essential amino acids and mineral composition in relation to either traditional (“ogi”) or commercial (Nutrend) complementary food for infants in Nigeria (Ijarotimi & Keshinro, 2012). Cashew kernels, microwaved and stored for 6 months under room temperature, were found to be free from pest infestation and lipid rancidity when compared with unprocessed kernels (Das et al., 2014). Ajith et al. (2015) demonstrated that the proximate composition of raw cashew nuts is significantly affected after adjusting for surrounding air temperature and moisture during storage. Processes involved in removing the testa from the kernel were found to eliminate phenolics (Griffin & Dean, 2017). The testa of cashew kernels is high in polyphenolic compounds (+)‐catechin and (‐)‐epicatechin, giving the testa‐containing nuts a special antioxidant and health‐promoting nutritional value (Trox et al., 2011). Olalekan‐Adeniran & Ogunwolu, (2018) found that honey‐coated kernels had higher nutritional value when compared with oven‐roasted kernels.

Traditionally, cashew nuts are sun dried (Ajith et al., 2015) and then stored before they are industrially and manually processed. Raw cashew nuts are dried to reduce deteriorating moisture at harvest, from 25% to 7% (Adeigbe et al., 2015; Dendena & Corsi, 2014; Gyedu‐Akoto et al., 2014), and this is achieved by exposure to direct sunlight. However, heat and light from the sun in association with oxygen lead to loss of sensitive nutrients, for example flavonoids (Ali et al., 2016), and depending on the intensity and duration, a change in the quality characteristics of cashew kernels is observed (Bai et al., 2017).

In northern Mozambique, the intact fruits (nuts and apple) are dried directly in the sun and both parts are separated by twisting. However, in the southern region, as in many other parts of the world, both parts are twisted and separated immediately at harvest, and the nuts are sun dried separately while the apples are freshly processed into juice or fermented into wine and/or distilled.

**FIGURE 1:**
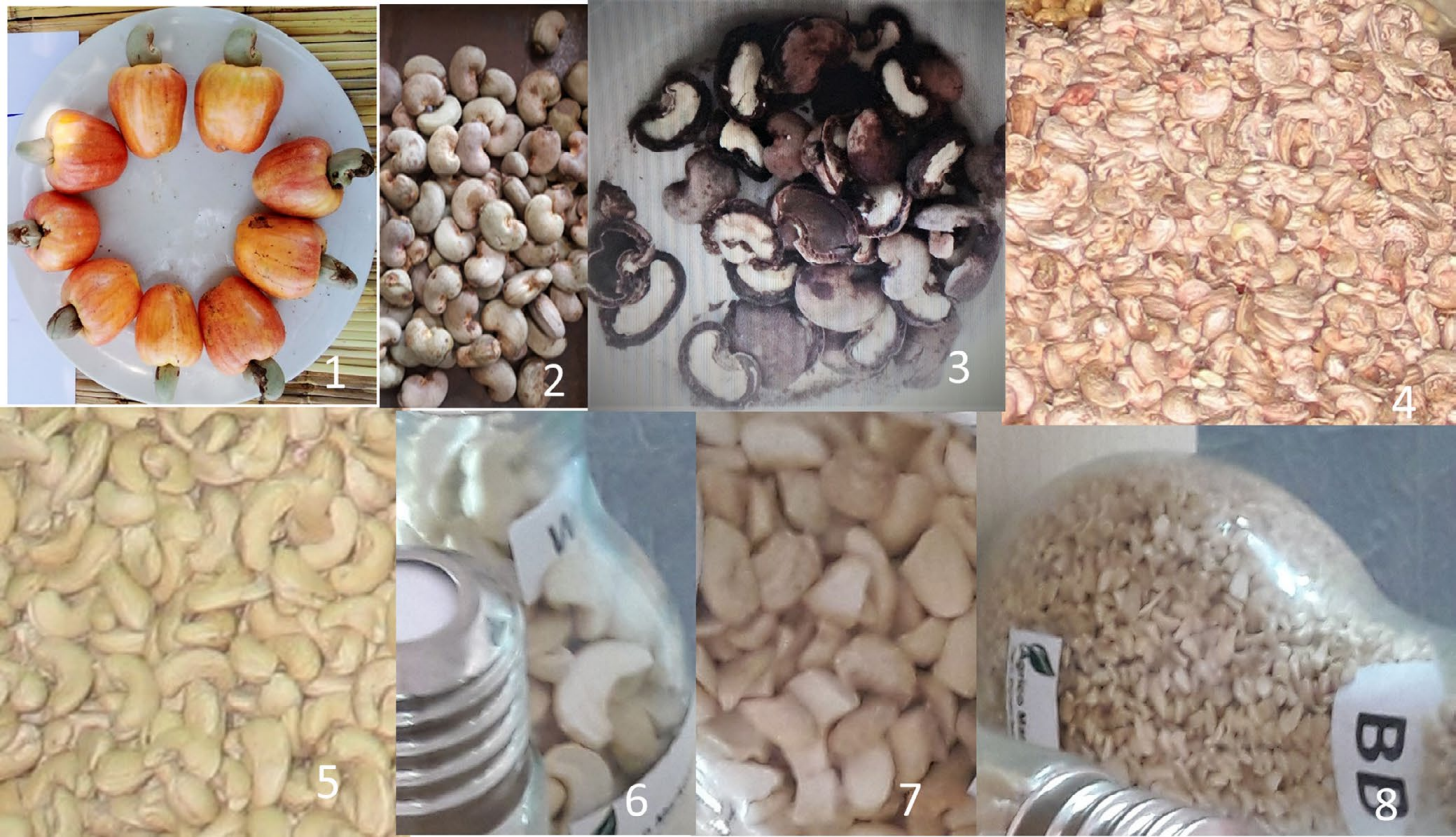
(1) Cashew nuts with apples—WCT‐A; (2) cashew nuts without apples—WCT; (3) hand‐cracked raw cashew nuts with white kernels inside—HC‐TC; (4) cashew kernels with testa—HC‐TC; (5) whole white cashew kernels without testa—WW; (6) cashew kernel splits—SP; (7) cashew kernel pieces (P); and (8) baby butts—BB

The key objective of industrial processing is to remove the kernel from the shell and separate the testa from the endocarp (Sunday & Abdulkarim, 2017). However, in this process, kernels can be broken, oversteamed, or damaged resulting in a series of different kernel grades based on their color, size, quality, and integrity. These categories are summarized (Figure 1) as white wholes (WW), splits (SP), P (pieces), and baby butts (BB) (De Carvalho et al., 2018) from which other subcategories are derived.

In a preliminary study (Uaciquete & Manjate, 2019), the proximate composition of total proteins, carbohydrates, ashes, and lipids was significantly different between the three grades, baby butts (BB), splits (SP), and white wholes (WW). Baby butts were found to be higher in proteins (22% versus 20.3%) and carbohydrates (33.8% versus 28.8%) when compared with splits and white wholes, which attests to the lower market price for the latter. In fact, many factors have been reported to influence the final nutritional quality of kernels: genotype (red or yellow), nut freshness, moisture content and postharvest processing, shelling methods, preshelling treatment, kernel grade, presence of testa, and the different roasting conditions (Akujobi et al., 2018; Bai et al., 2017; Ogunsina & Bamgboye, 2014; Trox et al., 2010, 2011). In the present study, we compared the nutritional profiles of carotenoids, tocols (tocopherols and tocotrienols), minerals (e.g., iron), fatty acids, and amino acids (1) of raw testa‐containing cashew kernels dried with or without the apple and (2) between wholes, splits, and baby butts of industrially steam boiled, processed, and oven‐dried nuts.

## 2 MATERIAL AND METHODS

### 2.1 Samples

#### 2.1.1 Cashew nuts

Samples were collected from the Nassuruma Cashew Research Center (14° 59’ and 38.162’’ South and 039° 42’ and 53.304’’ East), Mozambique, in the middle of the 2018/2019 cashew harvest season (November, 2018). Two sets were considered for the experiment: One consisted of nuts that were separated from the apples immediately after picking them from the ground. The second set was made of nuts that were picked with their respective apples joined together. The nuts without apples (WCT, Figure 1, plate2) were sun dried for 4 days on a sandy ground (mean daily temperature 33°C). The nuts with apples (WCT‐A, plate 1) were sun dried for 10 days, until the apples had dried (humidity < 10%) and were manually separated from their respective apples. Both sets of nuts were placed in jute bags and the bags were stored together in a common warehouse (mean daily temperature, 35°C). A sample of 4 kg was taken from each set, and cashew nuts were manually cut using nut‐cutting scissors to extract the kernels with the testa intact (HC‐TC, Figure 1, plates 3 and 4) and keeping minimum contact with shell liquid. About a kilo of cashew kernels was recovered from each set of cashew nuts, consisting of whole cashew kernels with testa, dried with the apple (WCT‐A) and cashew kernels with testa dried without the apple (WCT). Both sets were preserved with their respective testa in black polyethylene bags under room temperature with no direct light contact.

#### 2.1.2 Cashew kernel samples

Three sets of cashew kernels were obtained from Condor Nuts, a cashew nut – processing factory in Anchilo, Nampula. The nuts were harvested, during the crop season from October 2017 to January 2018. The cashew kernels were then processing as follows: nut steaming between 95 and 100°C and 4–5 bars of pressure, for 8–14 min, nut cracking for kernel extraction, kernel oven heating at 80°C for 8 hr, kernel cooling, and peeling or testa removal. Sample of different industrial grades—white wholes (WW, Figure 1, plate 5), splits (SP, plate 6), and baby butts (BB, plate 8)— were obtained from the factory in July 2018. All samples were preserved in polyethylene bags under normal room temperature. Finally, random aliquots of 250 g were used for the analysis of macro‐ and micronutrients.

#### 2.1.3 Preparation of the samples

Cashew kernel samples were stored at −80°C for 2 months until further processing. Samples were gently ground to fine powder under cooling with liquid nitrogen using an analytical mill (IKA A11 basic, IKA‐Werke, Staufen, Germany).

### 2.2 Analysis of macro‐ and micronutrients

#### 2.2.1 Carotenoids and tocols

Using HPLC, carotenoids (lutein, zeaxanthin, α‐/β‐carotene) and tocols (tocopherols and tocotrienols) were determined in accordance with previous research (Gowele et al., 2019; Grebenstein & Frank, 2012; Lux et al., 2020). In brief, saponified samples were extracted with n‐hexane, evaporated, and reconstituted with acetonitrile‐dioxane or ethanol in order to analyze carotenoids and tocols using UV–Vis and fluorescence detection, respectively.

#### 2.2.2 Minerals

Iron (Fe), zinc (Zn), calcium (Ca), magnesium (Mg), sodium, and phosphate contents were determined by inductively coupled plasma optical emission spectrometry (ICP‐OES) after microwave‐heated nitric acid digestion using an ultraclave, as previously described (Stuetz et al., 2012).

#### 2.2.3 Fatty acids

Fatty acids (FA) were determined by gas chromatography with mass spectrometry (GC/MS) after transesterification into methyl esters (FAMEs) using previously published protocols (Trox et al., 2010) with the following modifications. In brief, lipids were obtained by accelerated solvent extraction of approx. 1.5 g grounded sample in an ASE 350 apparatus (Thermo Fisher scientific, Waltham, MA, USA) (Weichbrodt et al., 2000). An aliquot of the lipid extract was used for gravimetric determination of the fat content, and another aliquot was transesterified by adding 1 ml of sulfuric methanol (1% H_2_SO_4_ acid in MeOH (*v*/*v*)) and incubating at 80°C for 1 hr. After washing and extraction into *n*‐hexane, FAMEs were analyzed by means of a 5890 series II Plus and 5972 GC/MS system in combination with a 7673 autosampler (all Hewlett‐Packard/Agilent, Waldbronn, Germany) in full scan mode (*m/z* 50–550). Analytes were separated on a fused‐silica capillary column (60 m x 0.25 mm i.d. coated with a 0.1‐µm film of 10% cyanopropylphenyl, 90% biscyanopropyl polysiloxane (Rtx‐2330, Restek, Bellefonte, PA, USA). Helium gas (99.999% purity) was used as the carrier with a flow rate of 1 ml/min and a GC oven program, which started at 60°C (held for 1 min); then, the temperature was raised at 6°C/min to 150°C, at 4°C/min to 190°C, and finally at 7°C/min to 250°C, which was held for 7 min; quantification was carried out in selected iron monitoring (SIM) mode (Thurnhofer et al., 2008).

#### 2.2.4 Amino acids

Amino acids (AA) in cashew nut samples were determined in accordance with commission regulation (EC) No 152/2009, annex III F: CELEX No 02009R0152‐20,130,212 (https://eur‐lex.europa.eu/eli/reg/2009/152/2013‐02‐12). Sample aliquots of 0.5 — 2 g were ground (size 0.75 — 1 mm), homogenized , and defatted (each sample aliquot with 150 ml of petroleum ether (b.r. 40–60°C)). An aliquot (about 1 g) was oxidized with performic acid–phenol mixture (at 0°C ice bath and 16 hr in a fridge) for the analysis of the sulfur‐containing cysteine/cystine and methionine, while tyrosine and other AA (except tryptophan) were analyzed in unoxidized samples. All samples were hydrolyzed with hydrochloric acid (6 M HCl containing 1 g phenol/L) at 110°C (mixture temperature) for 23 hr, separated by ion exchange chromatography (oxidized feedstuff column, type cation exchanger resin, 20 x 4.6 mm column), postcolumn‐derivatized with ninhydrin and photometrically detected (570 nm for primary AA and 440 nm for secondary AA, e.g., for proline) using an amino acid analyzer (Biochrom 30, Biochrom, Cambridge, England).

An aliquot (0.5—1 g of cashew kernel sample) was hydrolyzed under alkaline conditions (using barium hydroxide solution and heated to 125°C for 16 hr) and analyzed on tryptophan by HPLC (Agilent Technologies 1200 Series). The internal standard α‐methyl‐tryptophan was added, and tryptophan was separated and determined by use of a Nucleodur C18 Pyramid column (EC 125/4, 5 µm, Macherey‐Nagel) and fluorescence detection (EX: 280 nm, EM: 356 nm).

### 2.3 Statistical analysis

Concentrations of analyzed compounds were found to be normally distributed using the Kolmogorov–Smirnov test. Values were presented as mean (*SD*). Among whole cashew kernels with testa, those dried with apple (WCT‐A) were compared with those dried without the apple (WCT) using independent Student's *t* tests. In the second batch of samples, whole white kernels (WW) were compared with splits (SP, half kernels) and baby butts (BB) using ANOVA and Scheffe post hoc test. Two‐tailed *p* values of <0.05 were considered to be statistically significant; all statistical analyses were carried out using SPSS for Windows (SPSS, Chicago, IL, USA version 20.0).

## 3 RESULTS AND DISCUSSION

In the present study, carotenoids, tocols, minerals, fatty acids, and amino acids of (1) raw hand‐cracked testa‐containing cashew kernels (HC‐TK) dried with and without the apples and of (2) industrially processed kernels (IPK), and therefore testa‐free whole whites (WW), splits (SP), and baby butts (BB), were analyzed and evaluated.

### 3.1 Carotenoids

Among the testa‐containing raw kernels (HC‐TK), total carotenoids (64.3µg/100g versus 52.6 µg/100g) and lutein (45.7 versus 34.6µg/100g) were significantly higher in the kernels from conventionally dried nuts (WCT) than in those cashew kernels from nuts dried “with apples” (WCT‐A). Among the industrially processed testa‐free kernels (IPK), no significant differences in total carotenoids were detected, but there was a higher concentration of a higher β‐carotene (7.21 versus 5.75µg/100g and 5.52µg/100g) in the whole whites (WW) than in splits (SP) and baby butts (BB) (Table 1).

**TABLE 1:** Carotenoids, tocopherols, and tocotrienols in raw cashews with testa, and in whole cashews versus splits and baby butts

[µg or mg/100 g]	WCT‐A	WCT	*P*_1_	WW	SP	BB	*P*_2_
	HC‐TC			IPK			
Carotenoids, µg							
Lutein	34.6 (1.81)	45.7 (6.04)	0.038	20.6 (2.02)	18.6 (2.76)	19.3 (1.17)	0.514
Zeaxanthin	2.89 (0.11)	3.31 (0.41)	0.163	2.22 (0.30)	2.30 (0.31)	2.14 (0.22)	0.783
α‐Carotene	0.97 (0.17)	0.66 (0.06)	0.044	0.51 (0.10)	0.46 (0.14)	0.35 (0.07)	0.231
β‐Carotene	14.1 (0.79)	14.6 (1.07)	0.504	7.21 (0.48)^a^	5.74 (0.57)^b^	5.52 (0.31)^b^	0.009
Total carotenoids	52.6 (2.38)	64.3 (5.48)	0.027	30.6 (2.56)	27.1 (3.35)	27.3 (1.11)	0.242
Tocopherols, mg							
δ‐Tocopherol	0.301 (0.006)	0.264 (0.027)	0.082	0.304 (0.006)	0.294 (0.010)	0.283 (0.014)	0.124
γ‐Tocopherol	2.324 (0.209)	2.556 (0.305)	0.339	2.364 (0.132)	2.704 (0.401)	2.012 (0.200)	0.054
β‐Tocopherol	0.008 (0.002)	0.004 (0.001)	0.072	0.005 (0.002)	0.007 (0.001)	0.004 (0.001)	0.113
α‐Tocopherol	0.563 (0.058)	0.624 (0.062)	0.285	0.212^a^ (0.039)	0.270^a^ (0.018)	0.134^b^ (0.019)	0.003
Total tocopherols	3.196 (0.274)	3.448 (0.378)	0.403	2.885^a,b^ (0.160)	3.275^a^ (0.378)	2.433^b^ (0.210)	0.023
Tocotrienols, mg							
δ‐Tocotrienol	0.170 (0.008)	0.152 (0.005)	0.033	0.284^a,b^ (0.011)	0.322^a^ (0.049)	0.216^b^ (0.005)	0.012
γ‐Tocotrienol	0.061 (0.002)	0.074 (0.012)	0.149	0.058^a,b^ (0.004)	0.068^a^ (0.009)	0.051^b^ (0.002)	0.046
β‐Tocotrienol	0.029 (0.002)	0.018 (0.016)	0.280	0.027^a^ (0.005)	0.021^a,b^ (0.002)	0.017^b^ (0.001)	0.019
α‐Tocotrienol	0.099 (0.004)	0.075 (0.015)	0.061	0.100^a,b^ (0.010)	0.120^a^ (0.013)	0.078^b^ (0.009)	0.010
Total tocotrienol	0.360 (0.010)	0.319 (0.019)	0.029	0.470^a^ (0.026)	0.530^a^ (0.064)	0.363^b^ (0.012)	0.007

Note

Values are mean and standard deviation (in brackets). ***P*_1_**, *p*‐value of *t* test: WCT‐A versus WCT; ***P*_2_**_,_ *p*‐value of ANOVA (WW versus SP versus BB);

Post hoc Scheffe test: values within a row not sharing a common superscript letter (^a,b^) are significantly different at *p* <.05.

HC‐TK (cashew kernels with testa): **WCT‐A**, whole cashew kernels with testa, dried with apple; and **WCT**, cashew kernels with testa dried without the apple.

IPK (industrially processed kernels): **WW**, white wholes; **SP**, splits, and **BB**, baby butts.

Total carotenoids of Mozambican cashew kernels in the HC‐TK kernels (52.6‐64.3 μg/100 g) were almost double that in the IPK kernels (27.1‐30.6 μg/100 g) (Table 1). This is consistent with a previous study (Trox et al., 2011) where significantly higher β‐carotene (21.8µg versus. 8.9µg/100g), lutein (52.5µg versus. 29.2µg/100g) and total carotenoids (75.0 versus. 38.9µg/100g) were found in testa‐containing than in test‐free hand‐cracked Indonesian cashew kernels. Hand‐cracked cashew nuts contain valuable carotenoids including the provitamin A active α‐ and β‐carotene. Furthermore, these provide very high antioxidants in the form of catechins, all of which are present in the testa (Trox et al., 2011).

### 3.2 Tocopherols and tocotrienols

In HC‐TC and IPK, there were no differences in total tocopherols, but tocotrienol contents were slightly higher in IPK than in HC‐TC. Slightly higher concentrations of tocopherols, were observed in WCT than in WCT‐A (Table 1). Higher amounts of tocopherols in WCT suggest a less oxidative stress caused by oxygen and direct sun intensity during drying. WCT‐A nuts are probably more stressed due to attempted redistribution of nutrients between the apple and the nut. Tocopherol decreases in response to different stresses induced, among others, by high‐intensity light and oxygen (Lushchak & Semchuk, 2012) by which tocopherols are degraded to tocopherolchinon (Trox et al., 2010).

Total tocopherols were significantly higher in splits (SP) than in baby butts (BB) (3.27 vs. 2.43mg/100g). Baby butts consist mainly of embryos and exes, which are small and relatively superficial compared to SP, therefore explaining higher exposure to oxygen and light during processing and storage and thus lower tocopherol concentrations. The highest concentration of total tocopherols was found in the conventionally dried (WCT) kernels with testa (3.45mg/100g). The high concentration of catechins in the testa acting as antioxidants is the obvious reason for the much higher α‐tocopherol concentrations in HC‐TC than in IPK (Trox et al., 2011).

IPK kernels were obtained from a local industry and were stored for 1 year longer than HC‐TC, which further reduces vitamin E concentrations via oxidative degradation. Rico et al. (2015) reported slightly higher total tocopherols in cashew kernels, 5.80 mg/100g, than any of our findings (Table 1). The difference may be due to the fact that the different processes employed in this study contributed to tocopherol decomposition (Trox et al., 2010). In agreement with previous analyses in cashews, γ‐ followed by α‐tocopherol were the predominant tocopherol isomers in both HC‐TC and IPK (Stuetz et al., 2017; Trox et al., 2010, 2011). On the one hand, γ‐tocopherol is often the most prevalent form of vitamin E in plant seeds (Trox et al., 2010). On the other hand, α‐tocopherol has the highest antioxidant activity (Ciebiera et al., 2018) of all tocopherol isomers. In accordance with previous research (Nguyen Phuoc Minh et al., 2019; Stuetz et al., 2017), we found testa‐containing cashew nuts as the richest source of vitamin E.

Tocotrienols were found to be higher in IPK (0.36–0.53mg/100g) than in HC‐TK (0.32–0.36 mg/100g) samples and consisted mainly of δ‐tocotrienol. Tocotrienols account for 10%–15% of tocopherols, which is consistent with the study of (Hejtmánková et al. (2018), who found significantly lower quantities of tocotrienols than tocopherols in all dried nut types including cashew kernels. Both total tocotrienol and δ‐tocotrienol were significantly higher in WCT‐A than in WCT (Table 1). The stability of tocotrienols may be influenced by storage and processing (Shahidi & Camargo, 2016). Therefore, the observed higher content of tocotrienols in WCT‐A kernels may be due to the partial shading conferred by the apple during the drying process, thus avoiding heat‐triggered degradation of tocotrienols that is observed in conventionally dried WCT nuts.

Among the IPK, δ‐, γ‐, and α‐ tocotrienol were significantly higher (as shown for tocopherols) in splits than in baby butts. Splits is the kernel commercial term for the botanical cotyledons. That cotyledons contain more fat than embryos (Uaciquete & Manjate, 2019), explains the high concentrations of tocotrienols, as lipophilic molecules (Fiume et al., 2018), along with fats in splits. The splits also showed higher concentrations of tocotrienols than white wholes.

### 3.3 Minerals

The overall mineral profile of cashew nut kernels is shown in Table 2. High concentrations of magnesium, phosphorus, and calcium, as well as iron and zinc, of which the last two are especially important for human nutrition, are consistent with previous studies (Alonso et al., 2017; Rico et al., 2015).

**TABLE 2:** Minerals in raw cashew kernels with testa, and in whole cashews versus. splits and baby butts

Minerals [mg/100g]	WCT‐A	WCT	*P_1_*	WW	SP	BB	*P_2_*
	HC‐TC			IPK			
Iron	4.59 (0.05)	4.35 (0.08)	0.071	5.73^a,b^ (0.20)	5.35^b^ (0.17)	6.10^a^ (0.00)	0.036
Zinc	4.26 (0.08)	4.12 (0.02)	0.137	4.84^b^ (0.01)	4.95^a,b^ (0.17)	5.39^a^ (0.03)	0.024
Sodium	4.35 (0.21)	4.00 (0.09)	0.165	7.47 (1.19)	9.00 (0.12)	9.11 (0.07)	0.164
Calcium	33.4 (2.04)	31.5 (1.16)	0.370	35.5^b^ (2.86)	33.6^b^ (1.38)	47.3^a^ (0.66)	0.010
Magnesium	237 (7.78)	212 (3.54)	0.058	240^b^ (6.36)	235^b^ (4.24)	268^a^ (2.12)	0.010
Potassium	721 (38.2)	711 (4.95)	0.760	646 (10.6)	654 (9.19)	672 (10.6)	0.167
Phosphate	463 (6.36)	452 (7.07)	0.229	528 (17.0)	511 (11.3)	556 (5.66)	0.075

Note

Values are mean and standard deviation (in brackets). ***P*_1_**, *p*‐value of *t* test: WCT‐A versus WCT; ***P*_2_**_,_ *p*‐value of ANOVA (WW versus SP versus BB);

Post hoc Scheffe test: values within a row not sharing a common superscript letter (^a,b^) are significantly different at *p* <.05.

HC‐TK (cashew kernels with testa): **WCT‐A**, whole cashew kernels with testa, dried with apple; and **WCT**, cashew kernels with testa dried without the apple.

IPK (industrially processed kernels): **WW**, white wholes; **SP**, splits, and **BB**, baby butts.

WCT‐A nuts had higher concentrations of iron (4.59 versus 4.35mg/100g) and magnesium (236.5 versus. 212.5mg/100g) than kernels recovered from WCT nuts. In general, slightly higher concentrations of all analyzed minerals were observed in WCT‐A than in WCT. Phenology in cashew indicates that after the nut reaches its maximum size, it undergoes desiccation and shrinks and changes color from dark green to gray, while the, apple rapidly develops to reach the final size and undergoes maturity (Adiga et al., 2019). Nutrient flow in these processes is unknown. However, our present study suggests that apples may be still releasing minerals to the kernels in the late stages of maturation or postharvest. Further research comparing both apple and kernel mineral physiological relationships is needed.

In general, all IPK and, in particular, BB are somewhat higher in minerals than HC‐TK. Within IPK grades, all minerals were higher in baby butts category (BB) than in white wholes (WW) and/or splits (SP), containing significant amounts of magnesium, calcium, iron, and zinc. The higher mineral content is also the explanation for the significantly higher total ash in BB than in SP from our previous study (Uaciquete et al., 2017). IPK kernels in the present study were oven dried at 80°C for 24 hr and had the highest mineral content in BB. In general, the mineral content in baby butts is much higher due to the embryos, which contain many nutrients (Uaciquete & Manjate, 2019). In addition, moisture reduction by oven drying is very likely the reason for the generally higher mineral content in IPK than in HC‐TC; cashew kernels have been found to reduce moisture content from 4.57% in raw nuts to 3.27% in dry‐roasted nuts (Griffin & Dean, 2017). To our knowledge, this is the first study showing highest concentrations of essential iron, zinc, calcium, and magnesium in BB, which should justify an increase in their market value.

All minerals, except potassium, were found in slightly higher concentrations in IPK (WW, SP and BB) than in HC‐TK kernels (WCT‐A and WCT). Possible explanations include cultivars and cultivation area as the mineral profile of any plant species is highly dependent upon the mineral content and composition of the soil (Griffin & Dean, 2017).

Other factors such as climate environment and germplasm diversity (Rico et al., 2015) as well as the phytosanitary condition of the nuts (Uaciquete et al., 2017) may also influence mineral profile. However, this information could not be obtained in detail from the processing factory. Nuts are known to be a rich source of minerals such as iron, potassium, magnesium, and zinc (Innocent & Ugochukwu, 2013). Potassium was the most predominant mineral in Mozambican cashew kernels (672–721 mg/100 g), followed by phosphorus (452–456 mg/100g) and magnesium (212–268 mg/100 g), all playing a special role in cellular multiplication and differentiation in humans (Griffin & Dean, 2017). Iron and zinc were found to be in highest concentrations (6.1mg/100g and 5.4mg/100g, respectively) in BB category (Table 2). Thus, considering their potential role in human nutrition (Innocent & Ugochukwu, 2013), mineral‐rich, BB grade, cashew nuts would be the recommended choice.

### 3.4 Fatty acids

Oleic acid, linoleic acid, palmitic acid, and stearic acid were the predominant fatty acids in both HC‐TK and IPK (Table 3). Significantly higher concentrations of stearic acid (3.12 versus 3.02g/100g) and linoleic acid (11.05 versus. 9.72g/100g) were detected in WCT than in WCT‐A kernels. Cashew nut fruit formation in nature is stimulated by high temperature so that in a cashew tree canopy, the sunny side grows faster than the shady side (Waller et al., 1997). Thus, the slowing down on deposition of stearic and linoleic acids during the drying process may be attributed to the shading provided by the apple in WCT‐A nuts.

**TABLE 3:** Fatty acids in raw cashew kernels with testa, and in whole cashews versus splits and baby butts

Fatty acids [g/100 g]	WCT‐A (T1)	WCT (T2)	*P*_1_	WW (W1)	SP (splits)	BB (baby butts)	*P*_2_
	HC‐TK			IPK			
Myristic acid, 14:0	0.011 (0.004)	0.008 (0.000)	0.393	0.011 (0.003)	0.009 (0.000)	0.010 (0.001)	0.541
Pentadecanoic acid, 15:0	0.005 (0.000)	0.004 (0.000)	0.347	0.004 (0.000)	0.004 (0.000)	0.004 (0.000)	0.824
Palmitic acid, 16:0	3.877 (0.143)	3.831 (0.079)	0.728	3.896^b^ (0.116)	4.10^a,b^ (0.094)	4.325^a^ (0.039)	0.039
Margaric acid, 17:0	0.041 (0.002)	0.040 (0.002)	0.645	0.049 (0.005)	0.048 (0.001)	0.050 (0.003)	0.906
Stearic acid, 18:0	3.024 (0.026)	3.121 (0.006)	0.037	3.500^a^ (0.032)	3.032^b^ (0.086)	3.550^a^ (0.061)	0.007
Arachidic acid, 20:0	0.177 (0.006)	0.174 (0.000)	0.568	0.188^a^ (0.003)	0.165^b^ (0.000)	0.193^a^ (0.002)	0.001
Behenic acid, 22:0	0.018 (0.000)	0.017 (0.000)	0.043	0.017 (0.002)	0.015 (0.002)	0.018 (0.000)	0.287
Lignoceric acid, 24:0	0.012 (0.001)	0.013 (0.001)	0.225	0.016 (0.002)	0.013 (0.002)	0.016 (0.000)	0.335
Palmitoleic acid, 16:1	0.150 (0.005)	0.114 (0.005)	0.020	0.127^b^ (0.000)	0.152^a.b^ (0.000)	0.157^a^ (0.010)	0.030
Heptadecenoic acid, 17:1	0.013 (0.001)	0.012 (0.000)	0.195	0.016 (0.001)	0.016 (0.002)	0.016 (0.001)	0.786
Oleic acid, 18:1	24.60 (0.022)	24.62 (0.264)	0.912	24.14 (0.777)	23.79 (1.143)	24.79 (0.099)	0.524
Gondoic acid, 20:1	0.029 (0.002)	0.027 (0.001)	0.460	0.032 (0.004)	0.030 (0.001)	0.029 (0.000)	0.618
Linoleic acid, 18:2	9.724 (0.023)	11.05 (0.190)	0.010	11.06 (0.098)	10.89 (0.422)	11.15 (0.050)	0.631
Total fatty acids	41.68 (0.142)	43.03 (0.535)	0.075	43.05 (0.944)	42.26 (1.741)	44.31 (0.143)	0.334

Note

Values are mean and standard deviation (in brackets). ***P*_1_**, *p*‐value of *t* test: WCT‐A versus WCT; ***P*_2_**_,_ *p*‐value of ANOVA (WW versus SP versus BB);

Post hoc Scheffe test: values within a row not sharing a common superscript letter (^a,b^) are different at *p* <.05.

HC‐TK (cashew kernels with testa): **WCT‐A**, whole cashew kernels with testa, dried with apple; and **WCT**, cashew kernels with testa dried without the apple.

IPK (industrially processed kernels): **WW**, white wholes; **SP**, splits, and **BB**, baby butts.

Overall, there was no significant difference in fatty acids within the IPK and HC‐TC groups. Highest total FA were found in BB due to slightly higher amounts of oleic acid and linoleic acid. Linoleic acid has been found to increase in soy bean seeds when the producing plants are physiologically stressed due to soil moisture deficiency (Wijewardana et al., 2018). However, oleic acid and linoleic acid were the predominant fatty acids in the present samples, and the higher amounts in BB could be explained by higher concentrations in the embryos and thus supply for “later growth”. Significantly higher concentrations of palmitoleic acid, arachidic acid, stearic acid, and palmitic acid were also found in BB than in SP and/or WW. Palmitic acid can be provided in the diet or synthesized endogenously via de novo lipogenesis (Carta et al., 2017); therefore, it is not essential for human nutrition. Palmitoleic acid is produced almost exclusively through desaturation of palmitic acid via stearoyl‐CoA desaturase‐1. Although dietary sources of palmitoleic acid are very limited, it plays an important role in human lipogenesis as a “lipid‐controlling hormone” called lipokine, regulating systemic metabolism (Duckett et al., 2014). Arachidic acid and its metabolites facilitate wound healing (Tallima & Ridi, 2018).

Within the IPK, BB grade was higher in fatty acids and therefore a better source than WW and SP. This is the first report showing the profile of specific fatty acid distribution among different commercial cashew kernel grades. Most of the previous publications focused on the cashew kernel in relation to other nuts (Chung et al., 2013; Fiume et al., 2018; Griffin & Dean, 2017) or simply provided the proximate composition (Okonkwo & Ozoude, 2015) or provided profiles in derivatives such as oil (Kosoko et al., 2009; Liu et al., 2019), flour (Emelike et al., 2015; Ogungbenle & Afolayan, 2015) or butter (Jegede et al., 2020). In a preliminary study using the AOAC method (AOCS, 1983), total fat content was found to be significantly lower in BB grade (33.8 versus. 43.3%) when compared with splits (Uaciquete & Manjate, 2019). This is in contrast to the present results (44% in BB versus 42% in SP) and could be due not only to kernel variety but also to the methods used— gas chromatography and mass detection. In agreement with the present results, the predominant oleic acid and linoleic acid were previously determined in the range of 57%–62% and 18%–24% of the total fatty acids, respectively (Ghazzawi & Al‐Ismail, 2017).

Fatty acids in Mozambican cashew nut kernels were found to vary between 45 and 50g/10g (Rico et al., 2015). However, others reported 37g or 42g/100g (Freitas & Naves, 2010; Ogunwolu et al., 2015; Olalekan‐Adeniran & Ogunwolu, 2018). Oleic acid (23.8–24.8g/100g) and linoleic acid (9.7–11.2g/100g) were the most predominant unsaturated fatty acids, as previously reported by several other researchers (Liaotrakoon et al., 2016; Trox et al., 2010, 2011). Palmitic acid and stearic acid were overall lower than previously reported for Brazilian cashew nuts (Soares et al., 2013); however, palmitic acid and margaric, lignoceric, and heptadecenoic acids, which occur in very low concentrations, were found to be higher in IPK and here in BB than in HC‐TK. This is consistent with earlier studies, which attributed this to the better extractability of oils from IPK (Maria & Hannah, 2019; Salehi et al., 2019; Shahidi & Camargo, 2016).

Overall, palmitic acid, stearic acid, oleic acid, and linoleic acid were the most quantitatively and nutritionally remarkable fatty acids in both HC‐TK and IPK. Previous studies found a similar dominance of these fatty acids in cashew nuts and cashew oil (Liu et al., 2019; Soares et al., 2013; Trox et al., 2011). However, a detailed description of drying techniques and kernel categories provided in the present study is lacking in previous research.

### 3.5 Amino acids

Aspartic acid, glutamic acid, valine, leucine, and arginine were the predominant amino acids (>1.0g/100g) in both HC‐TC and IPK groups of kernels. Total amino acid content was higher in the IPK than in the HC‐TK group (Table 4). Besides a significant amount of serine, threonine, arginine, and total amino acids were higher in WCT than in WCT‐A. Serine can be synthesized in the human body by metabolism of excessive threonine and is needed for fat metabolism, cell membranes, muscle growth, and a healthy immune system (Serine (PubChem)). Threonine as an “essential amino acid” has to be supplied through diet and is involved in protein synthesis and required for the nervous system and fat metabolism (L‐Threonine (PubChem)). Conventionally, cashew nuts are supposed to be neatly detached from the apples before air drying them under the shade. This is meant to avoid fungal deterioration due to humidity from the apple and nutrient loss under direct sun drying (FOA Teca, 2012). However, in northern Mozambique, farmers dry the nuts with undetached apples to minimize theft. In addition, nuts are sun dried to speed up reduction in moisture content. However, the WCT‐A drying technology appeared to have no significant effect on the content of amino acids in the kernels.

**TABLE 4:** Amino acids [g/100 g] in raw cashews with testa, and in whole cashews versus splits and baby butts

Amino acids	WCT‐A	WCT	*P*_1_	WW	SP	BB	*P*_2_
	HC‐TK			IPK			
Aspartic acid	1.48 (0.03)	1.560 (0.05)	0.204	1.97 (0.033)	1.84 (0.08)	1.89 (0.09)	0.345
Threonine	0.60 (0.01)	0.63 (0.01)	0.051	0.73 (0.01)	0.73 (0.01)	0.75 (0.03)	0.542
Serine	0.85 (0.01)	0.91 (0.01)	0.039	1.12 (0.03)	1.12 (0.03)	1.16 (0.06)	0.576
Glutamic acid	2.82 (0.08)	2.74 (0.11)	0.517	3.19 (0.01)	3.25 (0.11)	3.27 (0.12)	0.719
Glycine	0.74 (0.02)	0.78 (0.03)	0.296	0.91 (0.01)	0.93 (0.03)	0.96 (0.05)	0.422
Alanine	0.68 (0.02)	0.71 (0.01)	0.300	0.86 (0.04)	0.85 (0.03)	0.87 (0.04)	0.878
Cysteine	0.34 (0.01)	0.33 (0.02)	0.592	0.39 (0.01)	0.39 (0.02)	0.39 (0.02)	1.000
Valine	1.62 (0.08)	1.73 (0.04)	0.243	2.10^b^ (0.04)	2.11^b^ (0.05)	2.41^a^ (0.03)	0.008
Methionine	0.31 (0.01)	0.29 (0.02)	0.493	0.36 (0.01)	0.37 (0.01)	0.36 (0.01)	0.740
Isoleucine	0.68 (0.03)	0.72 (0.04)	0.383	0.92 (0.05)	0.89 (0.02)	0.91 (0.02)	0.692
Leucine	1.21 (0.02)	1.28 (0.03)	0.122	1.59 (0.07)	1.54 (0.03)	1.58 (0.06)	0.728
Tyrosine	0.45 (0.01)	0.44 (0.06)	0.746	0.61 (0.00)	0.59 (0.00)	0.61 (0.01)	0.142
Phenylalanine	0.06 (0.03)	0.82 (0.03)	0.219	0.99 (0.04)	0.97 (0.03)	0.98 (0.05)	0.884
Histidine	0.38 (0.01)	0.40 (0.01)	0.293	0.48 (0.02)	0.47 (0.02)	0.48 (0.02)	0.868
Lysine	0.83 (0.02)	0.86 (0.03)	0.412	1.01 (0.04)	0.99 (0.03)	1.02 (0.04)	0.832
Arginine	1.66 (0.03)	1.79 (0.04)	0.069	2.34 (0.08)	2.26 (0.06)	2.38 (0.05)	0.335
Proline	0.66 (0.02)	0.71 (0.00)	0.095	0.83 (0.04)	0.81 (0.01)	0.82 (0.03)	0.881
Tryptophan	0.26 (0.00)	0.26 (0.01)	0.423	0.29 (0.00)	0.28 (0.01)	0.27 (0.01)	0.614
Total amino acids	16.39 (0.11)	16.99 (0.26)	0.095	20.71 (0.44)	20.44 (0.47)	21.17 (0.69)	0.490

Note

Values are mean and standard deviation (in brackets). ***P*_1_**, *p*‐value of *t* test: WCT‐A versus WCT; ***P_2_***, p‐value of ANOVA (WW versus SP versus BB);

Post hoc Scheffe test: values within a row not sharing a common superscript letter (^a,b^) are significantly different at *p* <.05.

HC‐TK (cashew kernels with testa): **WCT‐A**, whole cashew kernels with testa, dried with apple; and **WCT**, cashew kernels with testa dried without the apple.

IPK (industrially processed kernels): **WW**, white wholes; **SP**, splits, and **BB**, baby butts.

Within IPK, valine was the predominant amino acid in the BB category than in the WW and SP categories (2.42 versus 2.12 and 2.10g/100g, respectively) and is metabolized mainly by peripheral tissues such as muscles (Riazi et al., 2003). For plants in darkness, degradation of valine and other branched‐chain amino acids provides energy for germination and maintain amino acid homeostasis (Gipson et al., 2017), which is in line with the predominance in embryos of the BB category. In addition, valine‐glutamine proteins are known to form specific complexes with transcription factors involved in the regulation of defense against multiple pathogens as well as response to osmotic stress and extreme temperatures (León et al., 2021), which are relevant for safe germination and development of the seedling in the soil. Besides valine, other amino acids were predominant in BB than in WW and SP, as observed for minerals. This observation could help to strengthen the market price of the “undervalued” BB grade (Ogunsina & Bamgboye, 2014).

Total amino acids in cashew kernels were found to vary between 16.39 and 21.17g/100g, in agreement with an analysis on Brazilian cashew nuts (Freitas & Naves, 2010). Glutamic acid content was higher in Mozambican nuts (2.75–3.28g/100g) than in Indian cashew nuts (4.26g/100g; Chung et al., 2013). A previous study compared the chemical composition among different types of nuts and edible seeds with regard to nutrients and other bioactive compounds and related this composition to nutrition and health (Freitas & Naves, 2010). Concentrations and proportions of the other predominant amino acids arginine, valine, and aspartic acid have been confirmed by previous studies (Rico et al., 2015; Salehi et al., 2019).

The higher content of amino acids in IPK than in HC‐TK might be due to polypeptide hydrolysis and subsequent release of amino acids (Olatidoye et al., 2020). This is because normal industrial procedures such as steam boiling of nuts and the oven drying of kernels can release amino acids from kernels, making them more accessible for extraction and analysis.

In conclusion, two cashew nut drying methods (HC‐TK) and three industrially processed kernel grades (IPK) were analyzed and evaluated with regard to micronutrient profiles, namely carotenoids, tocopherols, tocotrienols, minerals, fatty acids, and amino acids. The results indicate that drying cashew kernels with the respective apple (WCT‐A) slightly decreased the content of some individual carotenoids, tocopherols, and amino acids but increased some tocotrienols and minerals (e.g., iron and magnesium) in relation to conventional direct sun drying (WCT). Further, we were also able to confirm that the carotenoids lutein and the provitamin A active β‐carotene in the “hand‐cracked” testa‐containing raw kernels (HC‐TK) were almost double the concentrations measured in the industrially processed kernel grades (IPK).

Regarding IPK, industrial grade baby butts was associated with lower concentrations in some carotenoids, tocopherols, and tocotrienols, but with significantly higher concentrations in most of the minerals and some fatty acids than in white wholes and splits. This is the first time that such nutritional characteristics are distinctively highlighted for this industrial category, thus contributing to the distinct role of baby butts in the cashew kernel market.

## CONFLICT OF INTEREST

The authors hereby declare no conflict of interest of any nature, including financial (patent, ownership, stock ownership, consultancies, speaker's fee), that would require disclosure with regard to publication of this article.

## AUTHOR CONTRIBUTIONS

**Neid Ali Ferreira:** Conceptualization (supporting); Data curation (equal); Formal analysis (equal); Investigation (supporting); Methodology (equal); Project administration (equal); Writing‐original draft (supporting). **Katja Lehnert:** Investigation (equal); Methodology (equal); Writing‐review & editing (equal). **Walter Vetter:** Investigation (equal); Methodology (equal); Supervision (supporting); Writing‐review & editing (equal). **Nadine Sus:** Formal analysis (equal); Investigation (equal); Methodology (equal). **Wolfgang Stuetz:** Formal analysis (equal); Investigation (equal); Methodology (equal); Software (lead); Writing‐original draft (equal); Writing‐review & editing (equal).

## Data Availability

The data that support the findings of this study are available from the corresponding author upon reasonable request.
